# Opportunities and challenges for synthetic biology in the therapy of inflammatory bowel disease

**DOI:** 10.3389/fbioe.2022.909591

**Published:** 2022-08-10

**Authors:** Yumeng Dong, Tiangang Xu, Guozheng Xiao, Ziyan Hu, Jingyu Chen

**Affiliations:** ^1^ College of Food Science and Nutritional Engineering, China Agricultural University, Beijing, China; ^2^ Suzhou U-Synbio Co., Ltd., Suzhou, China

**Keywords:** inflammatory bowel disease, MicroRNA, mesenchymal stem cell, live biotherapeutic products, fecal microbiota transplant, synthetic biology

## Abstract

Inflammatory bowel disease (IBD) is a complex, chronic intestinal inflammatory disorder that primarily includes Crohn’s disease (CD) and ulcerative colitis (UC). Although traditional antibiotics and immunosuppressants are known as the most effective and commonly used treatments, some limitations may be expected, such as limited efficacy in a small number of patients and gut flora disruption. A great many research studies have been done with respect to the etiology of IBD, while the composition of the gut microbiota is suggested as one of the most influential factors. Along with the development of synthetic biology and the continuing clarification of IBD etiology, broader prospects for novel approaches to IBD therapy could be obtained. This study presents an overview of the currently existing treatment options and possible therapeutic targets at the preclinical stage with respect to microbial synthesis technology in biological therapy. This study is highly correlated to the following topics: microbiota-derived metabolites, microRNAs, cell therapy, calreticulin, live biotherapeutic products (LBP), fecal microbiota transplantation (FMT), bacteriophages, engineered bacteria, and their functional secreted synthetic products for IBD medical implementation. Considering microorganisms as the main therapeutic component, as a result, the related clinical trial stability, effectiveness, and safety analysis may be the major challenges for upcoming research. This article strives to provide pharmaceutical researchers and developers with the most up-to-date information for adjuvant medicinal therapies based on synthetic biology.

## 1 Introduction

Inflammatory bowel disease (IBD), mainly including Crohn’s disease (CD) and ulcerative colitis (UC) is a complex, chronic intestinal autoimmune disease. Similar pathological changes and clinical symptoms are usually found in such diseases, while the targeting sites of inflammation may be varied in patients’ digestive systems. Inflammation caused by UC is mainly found in the colon or rectum, whereas inflammation caused by CD may be expected throughout the digestive tract, from the mouth to the anus (Zeng et al., 2019). In Western countries, the incidence of IBD stabilized in the 20th century, while the incidence rate in developing countries remains increased in recent years. For instance, the number of IBD patients is expected to reach 1.5 million by 2025 in China based on the current epidemiological data (Kaplan, 2015), which could place a significant strain on public health care systems worldwide. Aside from intervening in the development of IBD, investment in the investigation of IBD’s infectious causes and related novel treatment strategies could be the most efficient and economically friendly way to lessen the burden.

**FIGURE 1:**
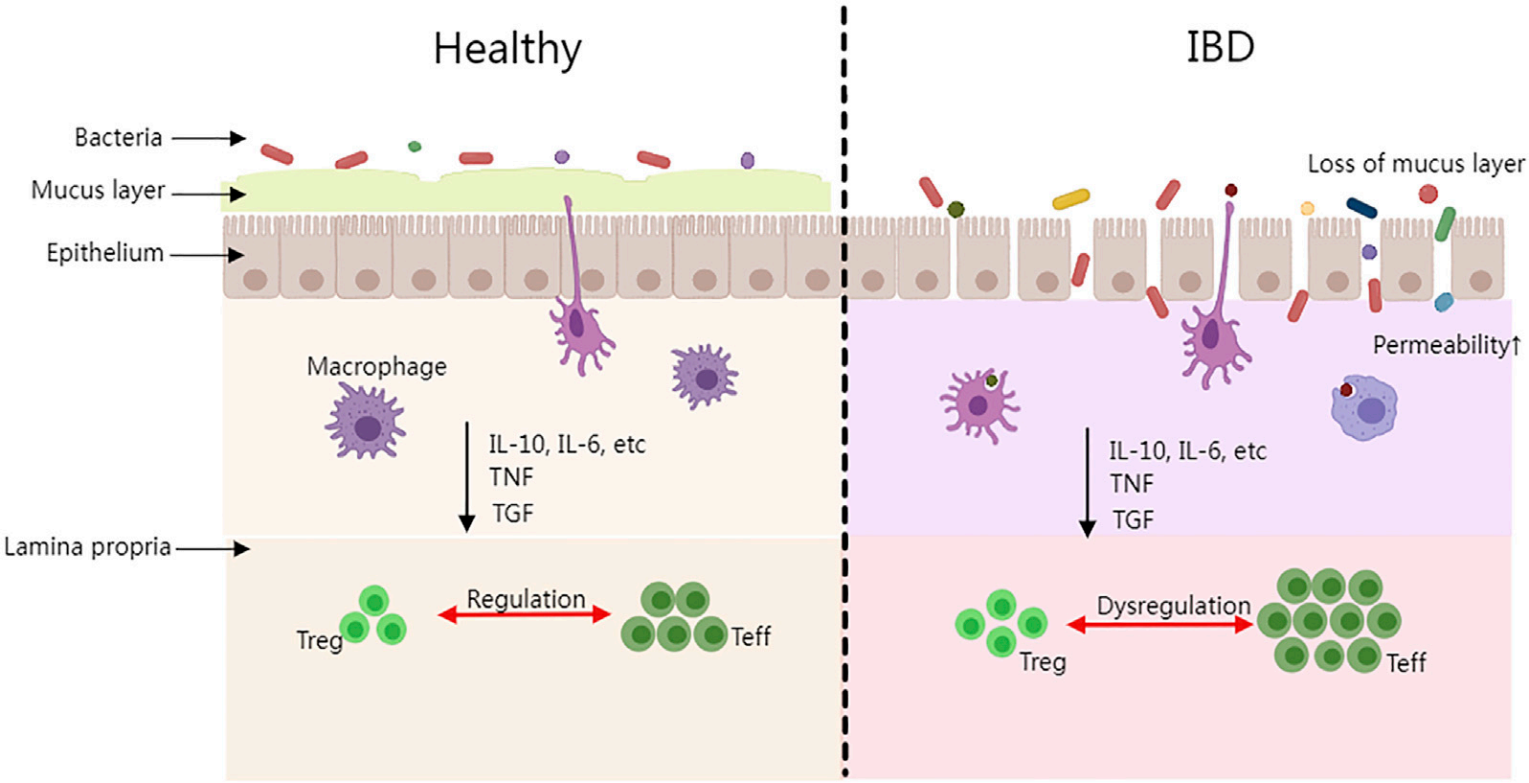
The intestinal difference between healthy people and IBD patients. (Treg, regulatory T cell; Teff, effector T cell; IL, interleukin; TNF, tumor necrosis factor; TGF, and transforming growth factor).

The pathogenesis of IBD is still under research, however, the dysbiosis of the gut microbiota, the host gene, the immune system, and non-inheritable factors are already known as the most prevalent consensus aspects. Firstly, the majority of IBD patients have imbalanced gut microbiota compositions. Intestinal species diversity and stability have dropped dramatically (mainly Firmicutes), while potentially dangerous microbes have increased (primarily Proteobacteria such as Enterobacteriaceae, Bilophila) and potentially protective anti-inflammatory microbes have declined (Lee and Chang, 2020). It is commonly acknowledged that the Firmicutes/Bacteroidetes (F/B) ratio plays a significant role in preserving a healthy intestinal homeostasis. Dysbiosis is characterized as a decreased F/B ratio, which is seen as a typical phenomenon for IBD (Stojanov, Berlec, and Štrukelj, 2020). In addition, Firmicutes and Bacteroidetes support the general adjustment of the intestinal mucus barrier (*in vivo*) by changing goblet cells and mucin glycosylation to maintain colonic epithelial homeostasis (Wrzosek et al., 2013). Secondly, polymorphisms and mutations of the host genome are essential factors in IBD development. Approximately 240 risk loci associated with IBD have been identified through genome-wide association studies, in which IBD is more susceptible among these groups of individuals (Lange et al., 2017). Thirdly, in IBD patients, the homeostatic balance of the immune system may be disrupted which could result in the induction of an inflammatory response. The complex regulatory process involves macrophages, dendritic cells (DC), helper T cells (Th), regulatory T cells (Treg), effector T cells (Teff), and other immune cells as well as cytokines, interleukin (IL), tumor necrosis factor (TNF), transforming growth factor (TGF), and so on secreted by immune cells (Rogler and Andus, 1998). IBD progresses and tissue damage is caused by the imbalance of pro-inflammatory and anti-inflammatory cytokines (Guan and Zhang, 2017). Figure 1 depicts the differences between healthy people with a balanced immune system and IBD patients in terms of epithelial tissue shape, microorganisms, and immunological components. Microbial antigens in the intestinal cavity migrate into the lamina propria when the intestinal epithelial barrier is breached. An acute mucosal inflammatory response is formed when immune cells in the intestinal lamina propria (such as macrophages and Teff) display a high immunological response and release a large number of cytokines (such as TNF, IL-10, IL-6, and TGF). The acute inflammatory response could stimulate the immune cells and help them eliminate germs and pathogens from the patient’s body. If immune cells continue to activate or the pathogens continue to stimulate the immune system while the activity of regulatory cells is suppressed during this process, chronic enteritis will develop over time, and persistent inflammation will lead to IBD disease. Finally, it is suggested that non-heritable factors such as environmental pollution factors, unbalanced daily diets, smoking, antibiotic abuse, etc., could play a role in IBD pathogenesis. Having a family history of IBD, nursing, eczema, and drinking tap water were all identified as risk factors in the incidence case investigations. The longer the breastfeeding period is, the larger the preventive effect it could have on the infant, thus lowering the risk of IBD. (Kane et al., 2000; Baron et al., 2005; Ananthakrishnan et al., 2018). Although, the aforementioned elements are all confirmed as related factors to the progression of IBD, it is known that none of the elements could be solely sufficient for IBD development.

**FIGURE 2:**
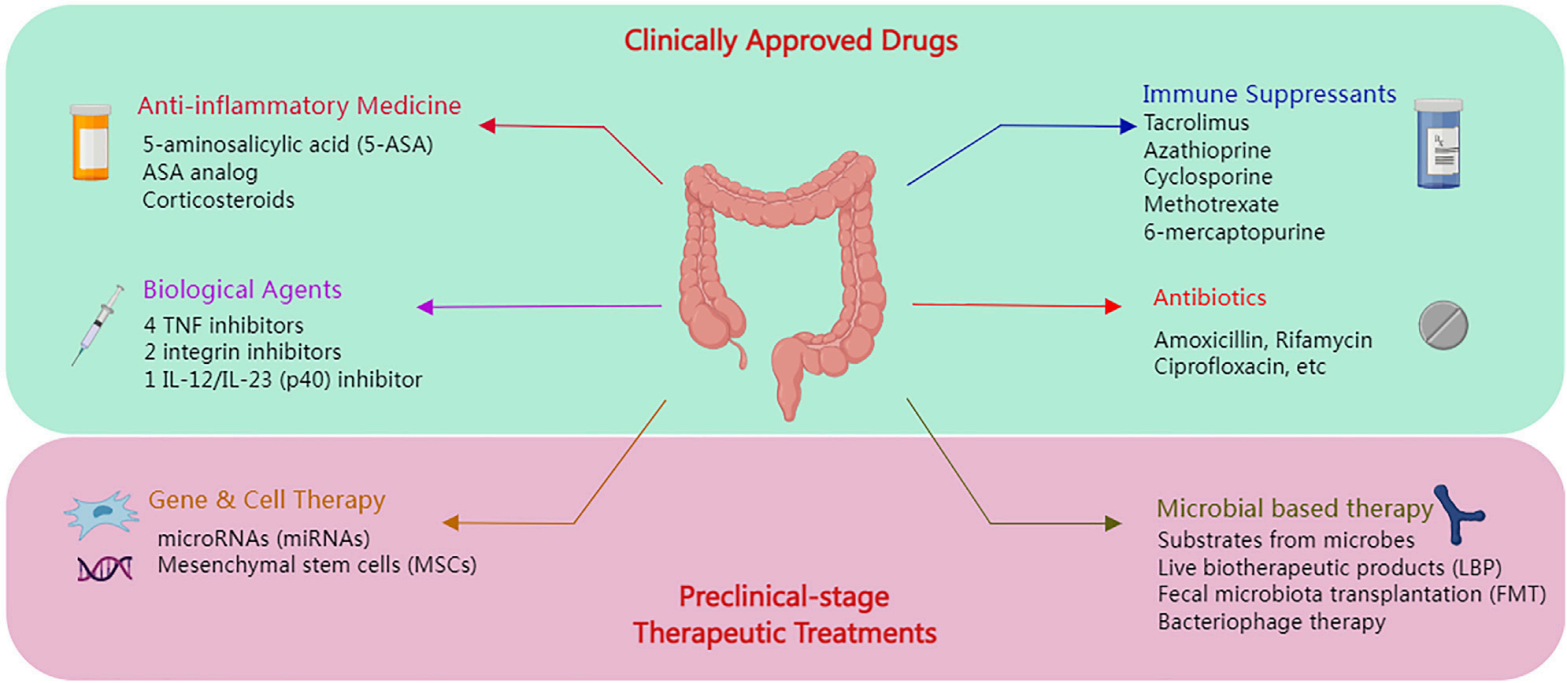
The overview of the IBD therapy treatment.

The therapeutic approach has also been developed at the same time as IBD pathological research, which suggested its clinically approved anti-inflammatory medications, antibiotics, corticosteroids, immune suppressants, and biological treatments. Meanwhile, an ongoing study also suggested further preclinical-stage therapeutic alternatives in genetic and cellular therapies, live biotherapeutic products (LBP), fecal microbiota transplantation (FMT), etc. (Oka and Sartor, 2020). Figure 2 displays the overview.

In the early 1940s, many drugs were developed to treat IBDs of various severity, such as mesalazine, olsalazine, and balsalazide disodium (Williams, 1994). Sulfasalazine (SAS) was one of the most significant and effective anti-inflammatory drugs; 5-aminosalicylic acid (5-ASA) is the active principle of SAS. Prednisone, budesonide, methylprednisolone, and hydrocortisone are among the corticosteroids used to treat UC (Bar-Meir et al., 1998; Schauer et al., 2021). Glucocorticoids have a long history in IBD treatment, which could help restore the intestinal barrier function as well as reduce inflammation (Riccardi et al., 2008; Greenhill, 2014; Marcin et al., 2016). Furthermore, previous literature also suggested evidence of glucocorticoid addiction, while some patients could be resistant to glucocorticoids. Moreover, IBD could also be treated with antibiotics such as amoxicillin, rifamycin, ciprofloxacin, ethambutol, and fosfomycin. Contrarily, antibiotics could result in the risk of decreasing the overall bacterial diversity while stifling beneficial bacteria, which might trigger an imbalance of the gut microbiota (Lewis et al., 2015). The immune suppressants, such as azathioprine, 6-mercaptopurine, methotrexate, cyclosporine, and tacrolimus, could be another type of treatment for IBD (Ardizzone, Cassinotti, and de Franchis, 2012). Other than that, biological agents could also be another alternative for IBD treatment. Regarding the treatment of inflammatory bowel disease (IBD), the Food and Drug Administration (FDA) has approved seven biologics in the current state of the art: four TNF inhibitors (infliximab, adalimumab, certolizumab, and golimumab); two integrin inhibitors (natalizumab and vedolizumab); and one inhibitor of IL-12/IL-23 (p40) (ustekinumab) (Ardizzone et al., 2012; Danese, Vuitton, and Peyrin-Biroulet, 2015). The pharmaceuticals nominated were approved clinically and authorized for sale. However, with the advancement of synthetic biology, efforts are being made to develop and deploy cutting-edge medical treatments.

Synthetic biology is a novel technology that combines computer science, molecular biology, system biology, bioengineering, and other interdisciplinary fields. It enables the creation of new biological substances or the restructuring of existing biosystems by modifying a genetic code or a critical metabolic pathway. Synthetic biology is progressing at a breakneck pace these days, due to the decline in the cost of biomolecular synthesis technology, the advanced progress of genetic engineering technology, and a profound grasp of genomic databases. The design and transformation of enzymes, metabolic pathways and networks, and biological chassis are at the core of synthetic biology technology. Currently, efficient enzyme design platforms based on synthetic biology have been built. Currently, effective platforms for creating enzymes have been built using synthetic biology. The rapid and targeted artificial evolution of enzymes has been carried out, the targeted enzyme mutants have been generated, and the enzyme libraries of various commonly used enzymes in the industry have successfully been constructed using high-throughput screening and testing methods. Simultaneously, the gene-editing platforms have been established to redesign the metabolic network of the chassis organisms, and a complete chassis cell bank including high-performance strains such as *Escherichia coli*, yeast, and lactic acid bacteria was established. Synthetic biology has had a significant impact on a variety of domains such as cell therapy (Yin et al., 2019), environmental pollution detection (Xinyi et al., 2019), the biosynthesis of unnatural compounds (Luo et al., 2019), and so on. Synthetic biology could also contribute greatly to the IBD treatment regarding various aspects, such as microbiota-derived metabolites, microRNAs (miRNAs), mesenchymal stem cells (MSCs), calreticulin, and microbe-related methods such as LBP, FMT, bacteriophage therapy, engineered bacteria, and their functional secreted synthetic products for IBD medical implementation.

## 2 Role of intestinal microbiota-derived metabolites in the therapy of IBD

As previously stated, the pathophysiology of IBD has not been fully researched. The relationship between intestinal microbes and innate immunity has been implicated in the etiology of IBD in several studies. However, the precise mechanism of action is still being investigated. Among the various pathogenesis mechanisms, the metabolites from the gut microbiota are considered to be one of the primary modes. The metabolites derived from different dietary substrates could influence the immune system and the permeability of the mucosal epithelium. The changes in the diversity and amount of the intestinal microbiota could lead to the fluctuation of the composition and concentration of metabolites. Among these metabolites, the bile acids, short-chain fatty acids (SCFAs), tryptophan, and succinic acid have been studied in the pathogenesis of IBD.

The bile acids produced in the liver are the end product of cholesterol metabolism. In addition to participating in the digestion of dietary lipids and fat-soluble vitamins, bile acids could also act as a signal regulator, exerting metabolic and immune effects (Marilidia et al., 2018; Albillos, Gottardi, and Rescigno, 2019). The bile acids are a group of molecules synthesized in the liver which can be further metabolized by the gut microbiota in the intestine. Multiple nuclear receptors are involved in the regulation of bile acid metabolism, such as the farnesoid X receptor (FXR), fibroblast growth factor 19 (FGF19), and G protein-coupled BAs receptor 5 (TGR5) (Browning et al., 2019; Q. Zhai et al., 2019a). The TGR5 regulates the macrophages by the nuclear factor- κB (NF-κB) and releases the cytokines IL-1, IL-6, and TNF (Calmus et al., 2010; Keitel et al., 2008; Y. D. Wang, et al., 2011). Through the regulation of the intestinal microbial composition and the gene expression of the corresponding regulatory factors, bile acid metabolism could be regulated, which implies that the bile acids could be a potential target in future IBD treatment.

SCFAs, which consist of acetate, propionate, and butyrate, are known as beneficial dietary metabolites generated from microbiota-accessible carbohydrates with different proportions depending on an individual’s dietary habit. The SCFAs could pass through the epithelium and trigger the transformation of Treg to Teff (Lavelle and Sokol, 2020). SCFA deficiency has been suggested by a previous research study as a risky influential factor in IBD development. Butyrate was suggested to have a boost effect on the butyrate transporter MCT-1 and reduce inflammation in UC patients *via* the inhibition of NF-kB activation (Vanhoutvin et al., 2009). The SCFAs are thought to be involved in the treatment of *Akkermansia muciniphila* (*A. muciniphila*) and *Clostridium* cocktails, which will be discussed in depth in the following part.

Tryptophan, an essential human aromatic amino acid, is obtained through everyday foods such as poultry and fish. The gut bacteria can convert tryptophan to aryl hydrocarbon receptor (AhR) ligands. T cell immunity is mediated by AhR, a transcription factor that is activated by IL-22 (Zenewicz et al., 2008). The AhR ligands were lowered and inflammation was reduced in mice after they were inoculated with three *Lactobacillus* strains that are capable of tryptophan metabolism (Lamas et al., 2016).

Aside from that, succinic acid, a tricarboxylic acid cycle intermediate, is becoming a hot topic in the treatment of IBD. Succinic acid has been shown to regulate macrophages *via* IL-1 (Mills et al., 2016).

In conclusion, research into the link between intestinal microbiota-derived metabolites and IBD has a promising future. Furthermore, future studies will focus on the regulation of microbiota-derived metabolites using a combination of metagenomics, host reporter assays, synthetic biology, and bioinformatics technology.

## 3 Role of miRNAs in the therapy of IBD

In the early 1990s, the non-coding single-stranded miRNA with a length of roughly 21–25 nucleotides was discovered. A great number of similar studies have been found on the critical role that miRNAs play in the onset and progression of IBD. Table 1 lists the recently discovered, abnormally expressed miRNAs in IBD patients, as well as their probable regulatory locations and signaling pathways. miRNA has been shown to affect key cytokines that are involved in the pathogenesis of IBD. Cytokines have a significant impact on regulating the immune response and maintaining physiological homeostasis. Immune illnesses like IBD could be caused by a cytokine imbalance. An in-depth study of the regulation process of cytokines is of great significance for the pathogenesis of IBD. In the future, predicting miRNAs that can regulate certain cytokines and better clarification of the regulatory mechanism of miRNAs against cytokines at the molecular level will be a new therapeutic drug development idea based on the biological roles of cytokines. Concerning the current research, miRNAs produced by host cells could infiltrate intestinal bacteria and thus could regulate the expression of intestinal bacterial genes, which enables a better management between the host and the intestinal bacteria. Gut microorganisms, on the other hand, could control the host gene expression by altering the level of expression of their miRNAs (Filip et al., 2016). In animal testing with a mouse model, inhibiting the expression of one of the miR-425 targets—Foxol was found to be able to interfere with the differentiation of T cell into Th17 (Xue et al., 2018). Blocking miRNA *in vivo* could be an appropriate therapeutic method for the treatment of IBD. Overall, sufficient literature studies have indicated the critical role miRNA plays in the diagnosis, prevention, and therapy of IBD.

**TABLE 1:** miRNAs and the targeted genes/signaling pathways/transcripts in IBD patients.

miRNA	Expression	Targeted gene/signaling pathway/transcript associated with IBD	References
miR-7	Increased	NF-κB signaling pathway; RNF183; IκBα	Qiao et al. (2016)
miR-10a	Decreased	MyD88 pathway; Toll-like receptor (TLR); Interleukin (IL)-12/IL-23p40	Wu et al. (2015)
miR-16	Decreased/Increased	NF-κB signaling pathway; denosine A2a receptor; TNF-α and IL-12p40	Huang et al. (2015); Tian et al. (2016)
miR-21	Increased	NF-κB signaling pathway; TNF-α; PTEN/PI3K/AKT pathway; RhoB; The programmed cell death 4 (PDCD4) tumor suppressor gene	Yang et al. (2013); Ludwig et al. (2013); Yang et al. (2013); Zhang, et al. (2015)
miR-31	Increased	WNT and Hippo signaling pathway; IL-13; IL-25; Hypoxia inducible factor 1	Markus et al. (2018), Olaru et al. (2015), Shi et al. (2017), Tian et al. (2019)
miR-23a	Increased	Tumor necrosis factor alpha inhibitor protein 3 (TNFαIP3); NF-κB signaling pathway; TNF-α	Felwick, et al. (2019)
miR-122	—	The gene nucleotide-binding oligomerization domain 2 (NOD2)	Chen et al. (2013)
miR-155	Increased	FOXO3a; IL13RA1; Est-1; IL-23/17/6	Min et al. (2014); Hou et al. (2017); Markus et al. (2018)
miR-185-3p	Increased	Colon cancer–associated transcript-1 (CCAT1); MLCK signaling pathway	Ma et al. (2019)
miR-192	Decreased	Macrophage Inflammatory Peptide-2α; NOD2	Chuang et al. (2014)
miR-214	Increased	NF-κB/IL-6 pathway; Phosphatase and tensin homolog; PDZ and LIM domain protein 2	Christos et al. (2015); Liu et al. (2019)
miR-141	Decreased	CXCL12β	Huang et al. (2014)
miR-494-3p	Decreased	IKKβ/NF-κB	Song et al. (2021)
miR-511-3p	Decreased/Increased	Toll-like receptor-4 (TLR4)	Heinsbroek et al. (2015)

The miRNA treatment method is not without its drawbacks. First and foremost, the efficient creation of miRNA differential expression profiles is required. MiRNA chip technology, gene set enrichment analysis, and bioinformatics analysis are currently being implemented to gradually settle the subject (Ma et al., 2019). Second, suitable targeted drug delivery vectors for miRNA nucleic acid medicines to intestinal immune cells must be explored and designed. Third, the future research plan is to evaluate the effect of the reproduction and blocking of the miRNA function on the abnormal response of the IBD immune system through cell and animal experiments. In summary, miRNA is expected to be an important gene target and a potential nucleic acid drug in the future.

## 4 Role of MSCs in the therapy of IBD

MSCs are known as one of the most commonly used stem cells in cell therapy, which have a promising and novel therapeutic future for the IBD treatment approach. MSCs’ excellent tissue regeneration and immune regulatory abilities provide a foundation for their broad application in IBD treatment. The efficacy and safety of low-dose MSC injections for CD therapy have been verified in recent trials (Molendijk et al., 2013). T cells, neutrophils, and macrophages are among the immune cells that MSCs can regulate (Wang et al., 2019). MSCs originating from various tissues, such as placenta (P-MSCs), umbilical cord (C-MSCs), bone marrow (bm-MSCs), adipose tissue (at-MSCs), and gingiva (g-MSCs) exhibit varying levels of proliferation, differentiation, and migration. MSCs from the placenta and adipose tissues have better immunoregulatory properties than the others (Talwadekar, et al., 2015). Furthermore, studies in mice showed that MSCs decrease IBD by changing the redox balance. In the MSC-injected animals, the levels of reactive oxygen species (ROS) and inflammation-related markers (TNF-, IL-4, and CD8) were reduced (Jung et al., 2020). Furthermore, a new MSC-coated approach shows that antibody-coated MSCs could be transported more efficiently to inflammatory colon regions, improving therapeutic efficacy. The survival rates of mice have improved considerably (Ko et al., 2010). In addition to immunosuppression and tissue repair ability, MSCs also have a strong effect on restoring the diversity and richness of normal gut flora, as well as on intestinal flora regulation. As a result, in the case of IBD, combining MSCs with microbial therapy could result in a more effective clinical therapeutic outcome. The *Lactobacillus* rhamnosus culture supernatant combined with bm-MSCs improves the intestinal barrier function and affects autophagy and lymphocyte function (Rui-Cong et al., 2016; Cai et al., 2019). However, there are still obstacles in the way of MSC therapeutic development: 1) the chemotactic mechanism of MSCs, as well as differentially expressed genes and pathways; 2) the efficient migration of MSCs to the targeted organs or tissues; 3) the *in vivo* residence period; and 4) the optimum source, dose, and infusion mode.

## 5 Role of calreticulin in the therapy of IBD

Calreticulin is a calcium-binding chaperone that has a function in integrin subunit activation (ITGAs). The suppression of calreticulin binding to ITGAs could reduce neutrophil and T cell adhesiveness, alleviating IBD symptoms. The interaction of the calreticulin and ITGAs on the pathogenesis of IBD, on the other hand, is currently being studied. ER-464195-01, a small oral chemical, was developed to prevent calreticulin from binding to ITGAs. Pro-inflammatory genes were downregulated and IBD’s severity was reduced in the mice models according to the transcriptome analysis (Ohkuro, Kim, Kuboi, Hayashi, and Fukamizu, 2018). Another study found that the mean level of anti-calreticulin antibodies was considerably higher in patients with UC than in people with healthy gut microbiota (Watanabe et al., 2006). Furthermore, Mendlovic et al. (2017) identified, cloned, and expressed the Taenia solium calreticulin. The experimental colitis mice were orally administered with the calreticulin. The calreticulin significantly reduced the inflammatory parameters, including TNF-α and IL-6, and thus prevented the experimental intestinal inflammation. Calreticulin has the potential to be used as a treatment for IBD related to immune suppressants and microbial-based medicine. The difficulties with this strategy are that it requires more research on the therapy mechanism. Synthetic biology could be used in the methodological development of transcriptomics or bioinformatics to find gene regulatory locations to control or block the calreticulin binding to its site of action.

## 6 Role of microbiota in the therapy of IBD

Intestinal microbial diversity and stability are essential variables in IBD. Furthermore, the loss of the mucus layer in IBD patients could increase the permeability of the epithelium to microorganisms, which could contribute to immunological activation and thereby induce an inflammatory response (Shan, Lee, and Chang, 2022). Based on microbial regulation, various therapeutic options may be available.

### 6.1 Live biotherapeutic products

Recently, the role of gut microbiota in the development, progression, and remission of IBD has caught much attention from pharmaceutical researchers and clinical product developers. Live bacterial species that may be able to survive and grow in the gastrointestinal tract and provide a health benefit to the host by modifying the microbiota are known as probiotics (FAO/WHO,2002). The LBP is regarded as the next-generation probiotic. LBP was defined by the FDA in 2016 as a biological product that: 1) contains live organisms, such as bacteria; 2) is used to prevent, treat, or cure a disease or a condition in humans; and 3) is not a vaccine. There has been preclinical research on the effectiveness of LBP in the treatment of IBD.

#### 6.1.1 *Lactococcus lactis* strains

*L. lactis* is a non-pathogenic, non-colonizing bacterium that has a long history of usage in fermented foods, which is classified as a “generally regarded as safe” (GRAS) microorganism by the FDA. *L. lactis* was genetically modified to produce biologically active compounds that could be administered directly to the mucosa. *L. lactis* was genetically modified to secrete the anti-inflammatory cytokine IL-10 by Steidler et al. (2000). In animal experiments, the daily administration of *L. lactis* expressing IL-10 resulted in a reduction in IBD symptoms. Another study suggested that *L. lactis* was modified to release elafin, which has anti-inflammatory characteristics. Elafin as a natural protease inhibitor is expressed in the healthy intestinal mucosa. In a mouse IBD model, the oral treatment of elafin-expressing *L. lactis* reduced inflammation and restored gut homeostasis (Motta et al., 2012). There was also an investigation into the ability of *L. lactis* I-1631 which is a non-engineered *L. lactis* isolated from fermented milk products to carry the bacterial enzyme superoxide dismutase (SodA) (Ballal et al., 2015). SodA has the ability to detoxify superoxide anions and show anti-oxidative characteristics. Because of the enzyme’s short half-life, SodA delivery by *L. lactis* would be more effective than SodA as an individual (Weber, 2015). Furthermore, a dairy *L. lactis* NZ9000 strain (NZ9000/IL-35) was modified to express murine IL-35. And an oral treatment of NZ9000/IL-35 inhibited the dextran sodium sulfate (DSS)-induced colitis progression. Furthermore, associated cytokines such as IL-6, IL-17, IFN-, and TNF- were proved to be modulated which suggested that NZ9000/IL-35 could be a good candidate for preventing IBD development (J. Wang et al., 2019). These investigations give an excellent foundation for the potential efficacy of engineered *L. lactis* as LBP in the treatment of IBD.

#### 6.1.2 *Saccharomyces* yeast strains

Since the 1950s, *Saccharomyces* yeast species have been authorized to be sold as probiotics and were categorized as safe strains (Mcfarland, 2010). Sylvester et al. (2012) conducted a study that showed that giving *Saccharomyces boulardii (S. boulardii)* to infants could be an effective treatment for necrotizing enterocolitis. However, another study suggested a contradictory outcome, which stated that an engineered *S. boulardii* producing IL-10 had no significant medical effect on IBD in mice as compared to the control group (Svenja et al., 2013). With the advancement of biotechnology, a yeast strain gene library was successfully constructed, which is useful for further designed editing. Furthermore, due to its increased efficacy, broader universality, and stability, the CRISPR-Cas9 system is gradually advancing in eukaryotic systems (Sen and Mansell, 2020). Scott et al. (2021) used a CRISPR–Cas9-based technique to create an altered *Saccharomyces cerevisiae (S.cerevisiae)* that expresses the human P2Y2 purinergic receptor and the ATP-degrading enzyme apyrase as a self-tunable probiotic yeast. The modified human P2Y2 receptor binds to eATP, which is produced by activated immune cells and commensal bacteria, with a 1,000-fold affinity. The eATP is thought to be an inflammation signal that promotes IBD progression by increasing the production of pro-inflammatory cytokines, inhibiting Treg activation, and raising the production of pro-inflammatory cytokines. In the meantime, the apyrase can hydrolyze eATP, reducing the inflammatory reaction. An oral administration of modified *S. cerevisiae* to mice reduced experimental intestinal inflammation (Scott et al., 2021).

#### 6.1.3 *Akkermansia muciniphila* strains

*A. muciniphila* is a next-generation probiotic that was first isolated from healthy human feces and has shown promise in the treatment of inflammatory bowel disease (IBD) (Derrien, 2004; Ting and Zhang, 2019). However, contradictory research results were obtained regarding whether *A. muciniphila* promotes or inhibits inflammation in IBD patients (T. Zhang et al., 2021). The positive results revealed: 1) the *A. muciniphila* probably relieves IBD by increasing SCFA production, improving the diversity of gut microbiota, and thus promoting Treg differentiation (Zhai R. et al., 2019); 2) the *A. muciniphila* probably relieves IBD by increasing SCFA production, improving the diversity of gut microbiota, and thus promoting Treg differentiation (Zhai et al., 2019); 3) In mouse research, *A. muciniphila* can successfully inhibit macrophage infiltration, hence blunting IBD (L. Wang et al., 2020). The negative results revealed: 1) *A. muciniphila* could allow microorganisms to enter the epithelium through mucus layer degradation (T. Zhang et al., 2021); 2) The *A. muciniphila* aggravated the symptoms of IBD in IL-10-deficient mice (Seregin et al., 2017), the mechanism is possible due to the lipopolysaccharides (LPS) of *A. muciniphila* which can cause higher levels of cytokine production including IL-1, IL-4, IL-6, TNF-α, etc. (Meng and Lowell, 1997; Singh and Jiang, 2003).

*A. muciniphila*, a type of gut commensal bacterium that could be utilized as LBP in the future, still has several limitations. The first step is to determine whether *A. muciniphila* has a pro-inflammatory or anti-inflammatory effect in IBD patients. Secondly, because *A. muciniphila* is particularly sensitive to oxygen, consideration must be given to its isolation, purification, cultivation, and storage (Ouwerkerk et al., 2016). Third, the mucin in the specific cultivation medium is an animal-derived protein that may cause an allergic response. Later, the use of *A. muciniphila* as an LBP could be concentrated on the aforementioned aspects.

#### 6.1.4 *Escherichia coli* strains

Since the first world war, *E. coli* Nissle 1917 (EcN) has been used in the treatment of numerous gastrointestinal disorders and is one of the best-studied non-pathogenic Gram-negative probiotic strains. EcN appeared to be as effective as mesalazine in the treatment of IBD, according to existing research and data obtained (Schultz and Butt, 2010). EcN was known as a popular carrier for the application of genetically engineered biosynthesis with respect to its qualities of safety and effectiveness. EcN was found to have a strong upregulation ability in the tight junction protein ZO-1 expression in murine intestinal epithelial cells in previous works. A higher ZO-1 expression protects mucosal permeability, and hence has the potential to be used as a treatment for IBD (Ukena et al., 2007).

EcN was also modified to release colicins such as E1 and E9, which have been found to kill adherent-invasive *E. coli* (AIEC) strains *via* an allelic exchange technique. The AIEC is thought to be a key pathogenic element in the development of IBD (Kotlowski, 2016).

#### 6.1.5 *Clostridium* cocktail

The *Clostridium* species could be classified into 19 clusters (I to XIX) (Collins et al., 1994). Based on previous studies, the *Clostridium* clusters IV, XIVa, and XVIII were decreased in IBD patients (Frank et al., 2007). Because different strains have varied metabolic and immunological activities, combining them could result in a more comprehensive IBD treatment effect. Atarashi et al. (2014) identified 17 *Clostridium* strains from healthy human feces and created a *Clostridium* cocktail to test the impact on IBD in mice. The results demonstrated that the cocktail can successfully prevent the intestinal inflammation caused by the DSS. The mechanism can be described by the following factors: 1) the synthesis of SCFAs, which elicits the Treg; 2) the conversion of indole from tryptophan by *Clostridium*, which has been shown to improve the epithelial barrier (Yosuke et al., 2013); and 3) a rise in gut microbial diversity.

### 6.2 Products from engineered microbes

#### 6.2.1 *Salmonella* effector protein AvrA

The soluble effector protein in intestinal probiotics transforms into the cytoplasm of the target cells and suppresses the inflammatory and immunoregulatory pathways, alleviating the IBD inflammation response. AvrA, a *Salmonella* acetyltransferase, inhibits the activation of a number of inflammatory effector genes. However, due to the pathogenicity of *Salmonella*, it is not appropriate to administer it. AvrA and other virulent proteins will be given together, potentially posing a health risk. As a result, using modern bioengineering technology, researchers discovered a strategy to solely distribute the naturally occurring immunomodulatory protein AvrA in the absence of *Salmonella*. The functional proteins were produced and purified after the ArvA genes were cloned in *E. coli*. Purified AvrA was then turned into a cross-linked protein nanoparticle that might be used to deliver drugs (Herrera Estrada et al., 2017). The anti-inflammatory efficacy was demonstrated *in vitro* and in murine colitis models, indicating that it has clinical promise for IBD treatment.

As molecular and immunology technologies advance, more molecular active compounds could be investigated for the treatment of IBD. Together with biotechnology, the intestinal effector pattern could lead to an effective therapeutic method in future IBD treatments.

### 6.3 Fecal microbiota transplantation

FMT has been used for more than 50 years since the discovery of the gut microbiota’s role in IBD. By engrafting the microbiota from a healthy donor, FMT aims to re-establish the gut microbial population in the recipient. After filtering, the donor’s feces were administered through enema, colonoscopy, nasoduodenal, or nasogastric infusion (Borody and Khoruts, 2012). Anderson et al. (2012) conducted a systemic analysis of FMT in IBD and found that 69% of patients with IBD were able to achieve remission. Greenhil. (2014) reviewed 31 studies that used FMT to treat IBD in which 71% of IBD patients reported a decrease in symptoms. The research available is minimal, and further work is needed to ensure that FMT is a valid strategy. The commercialization of the FMT approach faces a number of challenges, including an assurance of safety, contamination risk, donor stability, and public acceptance. Furthermore, the enteric virome must be considered because it has a significant impact on the host’s physiology. As a result, when performing the FMT treatment, it is vital to evaluate the potential risks posed by a change in the enteric virome (Norman et al., 2015). Furthermore, since the strains are long-term colonizers in the human gut, the FMT could be a trustworthy source of species for LBP development. The species isolated from the FMT preparations could also be considered safe cytokine delivery vehicles through microbial synthesis technology. The strains could help restructure the microbiome imbalances.

### 6.4 Bacteriophage therapy

Recently, the drug development research on bacteriophage therapy has returned to the fore again because of antibiotic resistance issues. According to the study, the number and abundance of bacteriophages on the surface of the intestinal mucosa have increased in IBD patients, implying that bacteriophages might display an undiscovered function in the progression of IBD. According to researchers, bacteriophages may kill probiotics in the colon, leading to a preponderance of “harmful bacteria” and an inflammatory reaction (Duerkop et al., 2018). To be more specific, the AIEC is thought to be a key pathogenic component in the development of IBD. Three phages targeting AIEC have been identified from wastewater and have been shown to diminish AIEC colonization in the intestine. In mice with DSS-induced colitis, the decrease of AIEC induced a laxative effect (Matthieu et al., 2017). Bacteriophages are a key component of the mucosal barrier’s defense against bacteria. Studies conducted *in vitro* have demonstrated that phages could stick to the mucus layer, reducing microbial colonization and disease (Barr et al., 2013). Additionally, research has shown that the development of metabolic diseases could be triggered by bacterial translocation from the gut to tissues, which would also cause inflammation. By directly eradicating fragile bacteria, phages could prevent bacterial translocation and, in return, the gut inflammation can be brought on by bacterial translocation (Qv et al., 2021).

The intricacy of intestinal bacteriophages and viruses, as well as their relationships, has increasingly been demonstrated using high-throughput metagenomic sequencing, transcriptomic, and proteomic techniques (Zwa et al., 2021). There are still many hurdles to overcome before bacteriophage therapy is approved for broad-scale clinical use: 1) scientific research and practical application need to be verified; 2) the virus database is not complete enough; 3) individual differences such as host age, sex, and diet; 4) the alteration of the bacteriophage community during IBD progression and the potential influential mechanism. In clinical experiments, bacteriophages could be utilized to target and destroy the bacteria that cause inflammation, perhaps slowing or even preventing the progression of IBD. Bacteriophage therapy could also be tailored to increase probiotic growth. Despite the fact that there are still many unknowns to be discovered, the promise of phage therapy is exciting. In the future, bacteriophage therapy could be considered to be used in conjunction with other microbial synthesis technologies to improve the specificity of virus pathogenicity and efficiently increase immunogenicity.

The existing and proposed therapy techniques based on microbial regulation are covered in the aforementioned text. Last but not least, the safety of genetically modified organisms (GMOs) is a serious challenge associated with the therapy approach based on genetically engineered bacteria. The implementation must be governed by strict regulations: 1) genetically engineered strains must be safe for human consumption; 2) the discharge and treatment of genetically engineered bacteria must be strictly regulated to avoid unpredictable gene variation, leakage, drift, and pollution; and 3) clinical applications must be subjected to a thorough scientific evaluation and strict government approval. In the current state of the art, the FDA’s attitude toward gene intervention therapy methods is to issue related regulations and guidelines (Jensen, Gtzsche, and Woldbye, 2021). In a nutshell, the benefits and drawbacks of microbial-based IBD therapy coexist.

**FIGURE 3:**
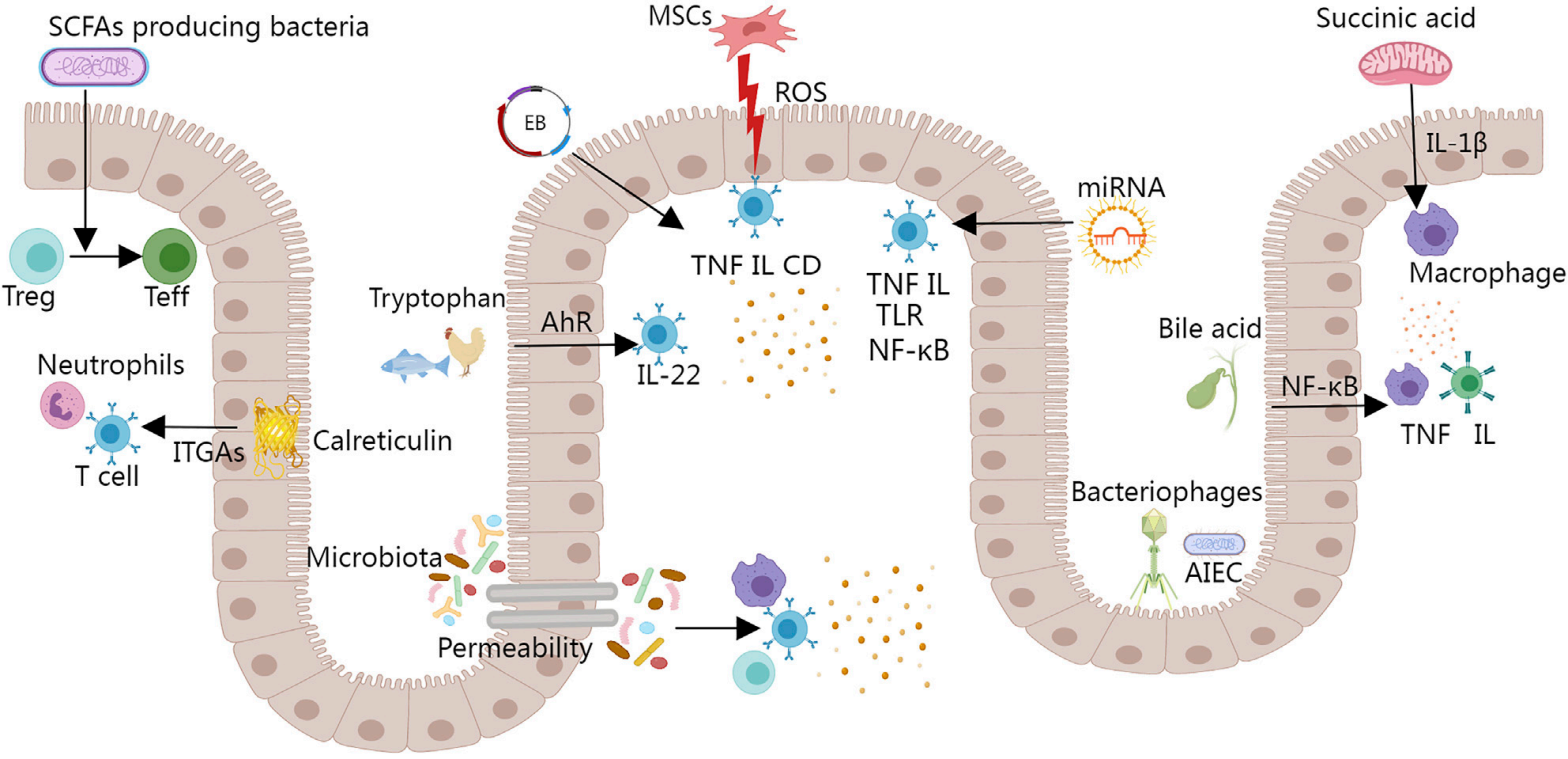
The current IBD treatment methods and potential targeted regulatory sites. (Treg, regulatory T cell; Teff, effector T cell; ITGAs, integrin α subunits; MSCs, mesenchymal stem cells; ROS, reactive oxygen species; EB, engineered bacteria; AhR, aryl hydrocarbon receptor; IL, interleukin; TNF, tumor necrosis factor; CD, cluster of differentiation; TLR, Toll-like receptor; NF-κB, nuclear factor- κB).

To date, IBD therapy remains a topic that requires more studies. It is still not exactly clear how the IBD pathogenesis is connected with gut microbiota, intestinal microbiota-derived metabolites, the immune system, etc. To elucidate the disease triggers in IBD, more *in vivo* and *in vitro* studies are required. With the finding of more therapeutic targets, further optimization of synthetic biology approaches may be needed in the future. The current popular therapeutic techniques, prospective targeted regulatory locations, and proposed mechanisms are outlined in Figure 3.

## 7 Conclusion

IBD, including CD and UC, is an autoimmune illness. With today’s fast-paced lifestyle, the number of IBD patients will continue to rise in the future due to unbalanced diets, work, and rest. The etiology of IBD is complex and the disease is easy to relapse, which brings a serious economic burden to patients and increases the pressure on society. Therefore, the ultimate purpose of the treatment of IBD is to reduce the number of relapses and hospitalizations, preserve long-term disease remission, and improve the long-term quality of life. Targeted antibiotic therapy is a sensible method, but the risk of resistant bacteria and the resulting gut flora imbalance make it unsuitable for long-term use. There is evidence showing that the imbalance of gut microbiota homeostasis is regarded as one of the essential initiating factors of IBD. Subsequently, the advancement of synthetic biotechnology provides technical assistance for new drug development.

The following procedures could offer suggestions for the clinical studies of brand-new medications: Firstly, the abnormal expression of genes, receptors, proteins, and other biomolecules in IBD patients was first evaluated using transcriptomics or protein analysis methods, as well as other biologic approaches. Secondly, pharmaceutical researchers and developers could create therapeutic strategies that are specifically targeted at the molecules with aberrant expressions. Thirdly, it is crucial to pick appropriate carriers for regulatory components. The options include engineering strains, encapsulation-coated techniques, MSCs, etc. Finally, to provide a theoretical basis for therapeutic application, research at the cellular and animal levels could also be conducted.

The FMT is a growing and appealing treatment, yet it still has a lot of flaws. Individual differences between donors and recipients, for example, are unknown, and there are no universal donors who can give consistent efficacy. Furthermore, there is no standardization of FMT donor selection, fecal sample preparation, or transplantation modality. And after the FMT therapy, the bacteriophages in parts of the patients increase. The increase of phages has the potential to intensify the inflammatory responses based on the animal study. But the stains isolated from the FMT could be further identified and applied to other biological treatment schemes.

In contrast, the LBP, as a next-generation product, is more flexible and easier to moderate. The LBP could be designed for specific patients based on the certainty of the microbial community of individuals. The specific gene could be operated by novel biotechnology and the microbes can be used as effective carriers. However, there are only therapeutic effects of the LBP in animal models and a finite proportion of IBD patients. More clear and meaningful research has not been found. This may be due to the genetic complexity associated with IBD and other environmental factors. In the future, with the advancement of clinical trials, LBP is believed to have great opportunities in the treatment of IBD.

Future drug development may consider multiple regulatory points and therapeutic approaches based on synthetic biology. To sum up, there are both opportunities and challenges for synthetic biology in the therapy of IBD. The challenges for the synthetic biotechnology-based therapeutic approaches are: 1) the deficiency in the study of the etiology mechanism of IBD; 2) the restriction of biotechnological implementation such as the completion of the related gene database; 3) the safety evaluation of live bacteria; 4) the efficacy and stability for long-term use including the passage cultivation analysis which is also an important index to evaluate whether the strain meets the needs of subsequent industrialization; and 5) the regulation of the novel drugs. There will still be a long way to keep on moving.
